# Detection of HER2 Gene Polymorphism in Breast Cancer: PCR Optimization Study

**DOI:** 10.3797/scipharm.ISP.2015.03

**Published:** 2016-02-14

**Authors:** Bugi Ratno Budiarto, Desriani

**Affiliations:** Research Center for Biotechnology, Indonesian Institute of Sciences (LIPI), Jalan Raya Bogor Km. 46, Cibinong 16911, Indonesia

**Keywords:** Breast cancer, HER2 gene, SNP, Allele-Specific PCR

## Abstract

Cancers are the most deadly diseases in the world and their incidences continue to increase over time. Particularly, breast cancer in females places 1^st^ rank among other types of cancers in term of cancer cases (23%) and death incidence (14%). Recent findings support the correlation between ^Ile^655^Val^ SNP in the HER2 gene with breast cancer risk. Moreover, the ^Ile^655^Val^ HER2 gene polymorphism could be a predictive factor in a neoadjuvant therapy setting. Precise detection of the ^Ile^655^Val^ HER2 gene SNP in early breast cancer patients will be beneficial in designing the most suitable treatment and in increasing the efficacy of anticancer drugs. Here we develop a rapid and inexpensive method for ^Ile^655^Val^ SNP detection in the HER2 gene based on allele-specific PCR technology. Two forward primers and one common reverse primer were designed to anneal specifically either on the HER2 gene fragment containing the GG genotype or to the HER2 gene fragment containing the AA genotype where one of these primers had been added with poly-GC at 5’ upstream. Moreover, to increase discrimination level, mismatch bases at the SNP site and the 3^rd^ base of each forward primers from 3’end were added. To test the performance of the designed primers in discriminating a polymorphism and its annealing temperature, breast cancer specimen-derived genomic DNA (with GG genotype) and pGEM_HER2/AA (with AA genotype) were used as templates in the PCR reaction. The optimal annealing temperature for SNP detection was at 51.5°C as showed by the appearance of a 150 base pair (bp) band as AA genotype (pGEM_HER2/AA template), 116bp band as GG genotype (genomic DNA template), and both types of bands as AG genotype (mix of pGEM_HER2/AA and genomic DNA template). Allelic types of breast cancer patients were also determined using this optimized method compared to sanger sequencing. The 100% accordance was shown for all types of genotypes in both methods. The allele-specific PCR in this study may have application in determining polymorphisms of the breast cancers-originated ^Ile^655^Val^ HER2 gene.

## Introduction

Breast cancer is one of common malignancy among women after cervical and ovary cancer and its incidence rate was 1.676.727 worldwide (http://www.cancerresearchuk.org). Surprisingly, in South-East Asia, Indonesia places the first rank for breast cancer incidence which is counted for 16.4% of national cancer incidence rate (http://www.cancerresearchuk.org). In accordance, many reports highlight the increasing trend in breast cancer incidence in developed countries and many of which have been detected in advanced stages where the cure is not possible [1]. Lifestyle (carcinogens, alcohol, tobacco, post-menopausal obesity and less exercise), reproductive factors (early menarche, late menopause, hormone level, and pregnancy) and diet with high-fat content have been established as the common risk factors for breast cancer development especially in asian population [2].

HER2 gene alteration has been associated with breast cancer development. Indeed, 10–40% of breast cancer incidence among women is caused by this gene alteration [3]. HER2 gene is a proto-oncogene with intrinsic tyrosine kinase activity located on chromosome 17q21, encoding a transmembrane glycoprotein which belongs to epidermal growth factors family [4]. Plenty of evidence have linked breast cancer risk with HER2 gene polymorphisms at codon 655, which is the conversion of amino acid isoleucine (**A**TC) to valine (**G**TC) [5–12]. It was assumed that this single amino acid change in human HER2 proteins mimics the biological role of rat’s mutated neu-protein (codon change at 664 position; valine into glutamic acid) in capacity to induce cells transformation through increasing the receptor dimerizations which impact on constitutive signaling activation [13]. Meanwhile other results have showed inverse results or null correlations occurred between them [14–16]. Nevertheless, since it was identified by Papewalis et al [17] 24 years ago, a polymorphism of HER2 at codon ^Ile^655^Val^ arises controversy among researchers worldwide until recently. Interesting finding published by Lu et al [18] who conducted comprehensive meta-analysis through SNP codon ^Ile^655^Val^ HER2 gene-related 27 published articles highlighted the dependency of ^Ile^655^Val^ HER2 polymorphisms as breast cancer risk factor to ethnicity.

Currently available methods for allelic types discrimination of HER2 gene polymorphisms of breast cancer patients are (1) PCR-RLFP and (2) Real-Time PCR using Taq Man-based or dual colour-based [17]. Two general steps are applied in PCR-RLFP where post-PCR treatment is mandatory [19]. After PCR reaction completed, the PCR product was digested with specific restriction enzymes such as *Bsm*AI that specifically recognize PCR-amplified DNA sequence for valine allele. Heterozygote allele (Val/Ile) appeared as two kinds of bands with different in size whereas Val/Val allele could be discriminated from Ile/Ile allele by simply looking at their size visually [5, 14]. Although PCR-RFLP is commonly used in genotyping of HER2 gene due to its simplicity and specificity, yet this method has several drawbacks such as a requirement for digesting the PCR products and visualizing the digested PCR products using gel electrophoresis so that the application of such method in a clinical setting is not only costly but also time-consuming. On the other hand, Taq-Man based Real Time PCR offers more precise and specific result for HER2 genotyping in breast cancer patients. Because of the success in genotyping using this method relies on probes and primers designed therefore some of technical notes must be seriously considered [6, 15]. Furthermore, using probes in this method will also further impact on experiment/clinical test cost. The methods with offering simplicity with low cost but keeping high specificity need to be developed to answer the clinical application need. Here, we develop the simplest PCR method to genotype SNP HER2 at codon 655 based on primers based-alleles discrimination with more simple, rapid, and specific result obtained.

## Results and Discussion

Current progress in breast cancer treatment highlights the importance of versatile biomarkers for diagnostic and prognostic application as well as for anticancer drugs therapy monitoring [20]. In many studies, it has been proven that HER2 polymorphism at ^IIe^655^Val^ codon has a strong association with breast cancer development in the case of HER2 protein overexpression and indeed those untreated patients with this protein abnormality showed worse clinical outcome [5] although some studies came with conflicting results as effect of sample size, methodoloical approacing, and source of samples collected [21]. Nevertherless, Cardiotoxicity-related transtuzumab treatment in breast cancer patients have been reported and this unwanted-side effect was observed in almost all of HER2 postive-breast cancer patients given standard treatment of transtuzumab. In this case, the cancer was strongly correlated with ^Ile^655^Val^ HER2 polymorphism and patients with Val allele significantly developed cardiac toxicity [22]. Both operable (44.7%) and metastatic breast cancers (41.9%) showed almost equal percentage event to have cardiotoxicity along transtuzumab therapy [23]. This fact indicates that (1) ^ile^655^Val^ HER2 polymorphism is a good biomarker in selecting patient group who is eligible to receipt transtuzumab without a high risk of developing cardiotoxicity and (2) the alternative therapies other than this treatment become more targeted and available to be used in group of suscesptible patients. Of 21 HER2 postive-patients breast cancer, we only detected 9.52% cases (allele specific PCR and sanger sequencing) with Ile/val genotype. Unfortunately, due to the limited clinical data records we have, the exact proportion for those patients who developed cardiotoxicity could not be accomplished. Furthermore, due to the limited sample currently available we also could not predict the real cardiotoxicity cases in this population. From the literature currently available, the number of cardiotoxicity cases for HER2-positive breast cancer patients with Ile/Val type obtaining transtuzumab therapy is almost 100% [22].

**Fig. 1:**
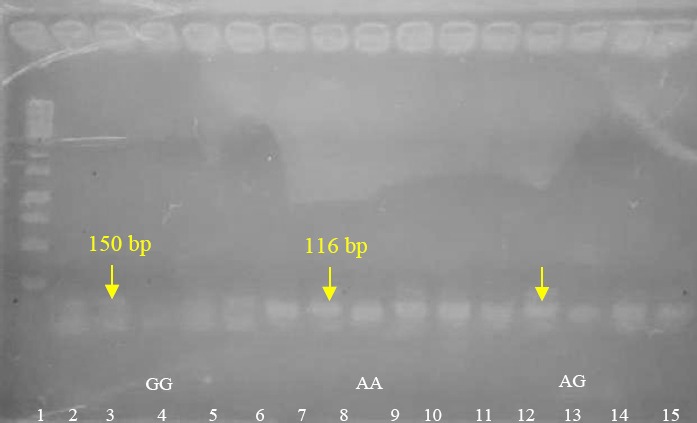
Gradient PCR results for determining the optimal annealing temperature used in HER2 genotyping of breast cancer patients. Lane 1 is 1 kb DNA ladder; lane 2 to 6 is PCR band for GG allele HER2 (PCR product size was 150 bp) ranging from 50 °C, 51.5 °C, 52 °C, 54 °C to 55 °C; lane 7 to 11 is PCR band for AA allele HER2 (PCR product size was 116 bp) ranging from 50 °C, 51.5 °C, 52 °C, 54 °C to 55 °C; lane 12 to 16 is PCR band for AG allele HER2 ranging from 50 °C, 51.5 °C, 52 °C, 54 °C to 55 °C. Yellow arrows indicated the optimum annealling temperature for allele specific PCR.

The key to support the success of ^Ile^655^Val^ HER2 polymorphism detection is how the method with high sensitivity and sensitivity selected and applied. In this study we applied allele-specific PCR to discriminate each allelic type of HER2 in breast cancer patients in a simple way using fragment sizes-based agarose electrophoresis. The discrimination power of allele-specific PCR for SNP detection solely depends on primers designed. Nucleotides composition, primers length, and GC content significantly impact on optimum annealing temperature of primers in PCR to amplify its DNA sequence target [24] and in the case of SNP genotyping the correct selection of mismatch base pairs in primer’s 3-end is also prerequisite [25]. Therefore, we firstly optimized the annealing temperature in which it must be able to clearly distinguish each allelic types of HER2 gene using known allele-template DNA. Applying temperature gradient ranged from 50°C up to 55°C in PCR process, each allelic types of HER2 gene was successfully discriminated at 51.5°C as pointed in figure 1. The AA genotype has PCR product size of 116 bp, The GG genotype has PCR product size of 150 bp, while AG mixed type harbors both of PCR product sizes. In our study, to increase the confidence and to avoid ambiguous in defining allelic types of HER2 gene, we applied two strategies which are (1) adding mismatch sites of SNP (A/G to T/G) and adding mismatch bases at 3^rd^ position from 3’terminal site of each forward primers and (2) adding poly GC bases in one of primer. These approaches seem to improve dramatically allele specificity just only judging on the differences in PCR product size [26.^27^]. The same strategy has also been successfully applied for genotyping 6 human SNP with high call rate (>98%), high accuracy (>99.9%) with minimal cost [28].

**Fig. 2:**
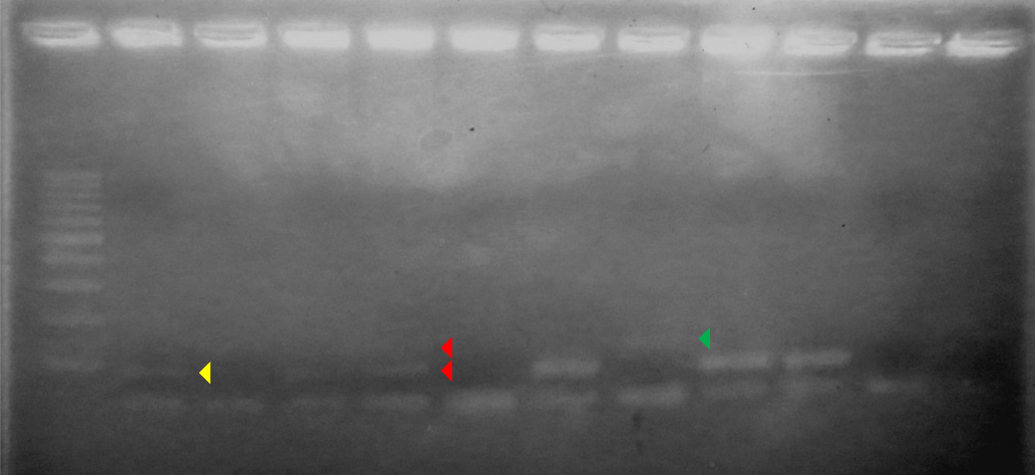
Allele-specific PCR representative for 10 of 21 samples of breast cancer patients. Yellow arrow head indicates AA genotype with band size of 116 bp; green arrow head indicates GG genotype with band size of 150 bp; red arrows head indicates AG genotype represented two different band sizes which were 116 bp and 150 bp respectively. The PCR product was run on 2.5% agarose under UV illumination. Lane 1 is 100 bp DNA ladder meanwhile NTC (Non-template control) was pointed in lane 12.

To validate SNP genotyping of HER2 gene using allele-specific PCR, we compared the band size pattern of PCR products with Sanger sequencing as a gold standard method used in clinical diagnostic. All allelic types of HER2 gene from breast cancer patients have been well discriminated using allele-specific PCR as shown in figure 2. Moreover, the perfect accordance occurred between both methods for all genotypes in the samples tested (table 1). This result shows that allele-specific PCR in our study has similar accuracy as to sanger sequencing was done. Therefore, our method could be potentially applied for determining polymorphism of breast cancers-originated ^Ile^655^Val^ HER2 gene in minimally equipped-lab setting or in a clinical setting with minimal cost.

**Tab. 1:**
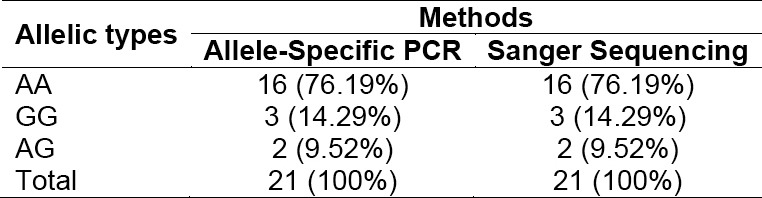
Comparison of allele-specific PCR with sanger sequencing of 21 breast cancer patients

## Experimental

### Sample

In this study, we extracted genomic DNA from selected 21 frozen section samples of archived biopsies of breast cancer patients whose HER2 status were positive. Samples were collected from M. Djamil Hospital Padang, Sumatera Province. Genomic DNA was isolated according to the manual tissue DNA extraction protocol (Pure Link Genomic DNA Mini Kit; Invitrogen). Meanwhile, pGEM_HER2_AA was obtained from *E. coli* DH5α recombinant using High Speed Plamid mini Kit (Geneaid) according to manual instruction. Involvement of subjects enrolled in this study has been approved by local ethics committee issued by Ministry of Health, Republic of Indonesia.

### Gradient PCR to determine the optimum annealing temperature for discriminating HER2 allelic types

The optimum annealing temperature for each HER2 alleles was determined using plasmid containing AA allele HER2 gene (pGEM_HER2_AA) and genomic DNA of GG allele HER2 positive-breast cancer in which its allele type was previously tested using sanger sequencing. Each 25 µL reaction mixture contained 12.5 µL of PCR Super Mix (Invitrogen), 10 pmol of allele-specific forward primer (5’-CCAGCCCTCTGACGTCCAGCT-3’), 10 pmol of long allele-specific forward primer (5’-GCGGGCAGGGCGGCGGGGGCGGGGCCCCAGCCCTCTGACGTCCACCG-3’), 10 pmol of reverse primer (5’-CGTGTACTTCCGGATCTTCTGCTG-3’), 10 ng of each DNA template. The PCR amplification condition was as follows: initial denaturation at 95 °C for 5 minutes, followed by 35 cycles of denaturation at 95 °C for 30 seconds, gradient temperature annealing from 50 °C, 51.5 °C, 52 °C, 54 °C to 55 °C for 60 seconds, and extension at 72 °C for 30 seconds (Kyratec Super Cycler Thermal Cycler, Australia). The PCR products were confirmed by running on 2.5% agarose gel and visualized under ultraviolet light.

### Allele-specific PCR 

Twenty one samples of breast cancer were tested for their allelic types using allele-specific PCR. The PCR condition and temperature profile was the same as mentioned above except for annealing temperature was fixed at 51.5 °C. The PCR products were confirmed by running them on 2.5% agarose gel and visualized under ultraviolet light. Sample which failed to be genotpyed then again repeated PCR. To prevent false-positive result, all PCR tubes and pipettes were pre-treated by exposing them on UV-light for ±15 minute prior to use. All PCR reagent mixing was done under laminar air flow.

### Sanger sequencing

Twenty one samples of breast cancer patients was amplified by conventional PCR using primer pairs HER2_F (5’-CCAGCCCTCTGACGTCCAT-3’) and HER2_R (5’-TCCGTTTCCTGCAGCAGTCTCC-3’) generating 142bp PCR product. The PCR amplification condition was as follows: initial denaturation at 95 °C for 5 minutes, followed by 35 cycles of denaturation at 95 °C for 30 seconds, anealling temperature at 60 °C for 30 seconds, and extension at 72 °C for 30 seconds. This PCR product was sequenced using HER2_R primer only done by First-Base Asia Ltd.
