# Correction: Budiarto, Bugi Ratno and Desriani. Detection of HER2 Gene Polymorphism in Breast Cancer: PCR Optimization Study. *Sci. Pharm.* 2016, *84*, 103–111

**DOI:** 10.3390/scipharm84040753

**Published:** 2016-12-02

**Authors:** Bugi Ratno Budiarto, Desriani

**Affiliations:** Research Center for Biotechnology, Indonesian Institute of Sciences (LIPI), Jalan Raya Bogor Km. 46, Cibinong 16911, Indonesia

The authors wish to make the following corrections to their paper [1]:

The primer sequence that previously appeared on page 108 as 5’-CCAGCCCTCTGACGTCCAGCT-3’, should be 5’-CCAGCCCTCTGACGTCCAGCA-3’.

The drug name transtuzumab should have been written as trastuzumab.

The sentence that appears on page 106 as“adding mismatch site of SNP (A/G to T/G)”, should have been written as“adding mismatch site of SNP (A/G to G/A)”.

The authors would like to apologize for any inconvenience caused to the readers by these changes.The change does not affect the scientific results. The manuscript will be updated and the original will remain online on the article webpage.
